# Antimicrobial susceptibility and genotyping of *Mycoplasma pneumoniae* isolates in Beijing, China, from 2014 to 2016

**DOI:** 10.1186/s13756-019-0469-7

**Published:** 2019-01-24

**Authors:** Fei Zhao, Jinrong Liu, Weixian Shi, Fang Huang, Liyong Liu, Shunying Zhao, Jianzhong Zhang

**Affiliations:** 1 National Institute for Communicable Disease Control and Prevention, Chinese Center for Disease Control and Prevention, 155 Changbai Road, Changping District, Beijing, 102206 China; 2 State Key Laboratory of Infectious Disease Prevention and Control, 155 Changbai Road, Changping District, Beijing, 102206 China; 3 Department of Respiratory Medicine, Beijing Children’s Hospital, National Center for Children’s Health, Capital Medical University, Nanlishi Road 56, Xicheng District, Beijing, China; 4 Beijing Center for Disease Control and Prevention, 16 Hepingli Middle Street, Dongcheng District, Beijing, 100013 China

**Keywords:** *Mycoplasma pneumoniae*, Macrolide resistance, 23S rRNA gene, Genotype

## Abstract

**Background:**

The presence of macrolide-resistant *Myocplasma pneumoniae* has been frequently reported in recent years, especially in China. In this study, we investigated the antimicrobial susceptibility and genotype against *M. pneumoniae* isolates from 2014 to 2016, Beijing.

**Methods:**

We investigated the activities of four antibiotics against 81 *M. pneumoniae* isolates in vitro*.* All isolates were amplification of domains II and V of the 23S rRNA gene and the L4 and L22 ribosomal protein fragments. All isolates were genotyped with duplex real-time PCR, MLVA and VNTR detection in p1 gene.

**Results:**

The macrolide resistance rate was 65.4% (53/81). Each of the macrolide-resistant *M. pneumoniae* isolates was resistant to erythromycin (Minimum Inhibitory Concentration, MIC, ≥256 μg/ml) and azithromycin (MIC, 2–64 μg/ml), but susceptible to tetracycline and levofloxacin in vitro. Fifty two macrolide-resistant isolates harbored the A2063G mutation, and only 1 macrolide-resistant isolates harbored the A2064G mutation in domain V of the 23S ribosomal RNA gene. The C162A, A430G, and T279C mutations in the L4 and L22 ribosomal protein genes were not responsible for macrolide resistance, but they were related to the particular genotype of *M. pneumoniae*. 95.7% of type 1 isolates (45/47) were macrolide-resistance, and 23.5% of the type 2 isolates (8/34) were macrolide-resistance. Type 2 *M. pneumoniae* macrolide-resistance rate was 50.6% higher than that of the previous reports in China. The eight macrolide-resistant type 2 *M. pneumoniae* isolates were belong to 3/5/6/2 and 3/5/7/2 MLVA genotypes.

**Conclusion:**

To our knowledge, this phenomenon likely resulted from a combination of genotype shifting from type1 to type 2 and antibiotic selection pressure in *M. pneumoniae* in China in recent years. The increase of resistance in type 2 is not due to the spread of same clone. However, the relationship between genotype shifts and macrolide resistance in *M. pneumoniae* needs to be further verified with more extensive surveillance data.

## Background

*Mycoplasma pneumoniae* is a common pathogen that causes human respiratory tract infections, especially in community-acquired pneumonia, which accounts for 10–40% of cases [1–3]. Although most of the infections are mild or self-limited, severe clinical symptoms are increasingly occurring. Furthermore, *M. pneumoniae* infections may lead to more severe extra-pulmonary conditions, such as erythema multiforme, myocarditis, and meningitis neuritis [4–6]. It is well known that *M. pneumoniae* is one of the smallest self-replicating organisms and lacks a cell wall, which makes it non-susceptible to β-lactam antibiotics. Therefore, macrolides are the primary drugs of choice for the treatment of *M. pneumoniae* infections in clinical settings [7].

Excessive or inappropriate use of antibiotics provides selective pressure for the development of macrolide resistance in *M. pneumoniae*. Since 2000, the emergence of macrolide-resistant *M. pneumoniae* strains has become a growing global problem. Macrolide resistance rates were reported to be approximately 3% in Germany [8], 10% in France [9], 2% in Switzerland [10], 26% in Italy [11], 3% in Denmark [12], and 11% in the United States [13]. Finally in Spain, the presence of a macrolide-resistant *M. pneumoniae* isolate causing community-acquired pneumonia was first reported in 2014 [14]. Although Europe and the United States have lower rates of macrolide resistance, Asiatic countries have the highest rates. After the first macrolide-resistant strain of *M. pneumoniae* was isolated in 2001 [15], Japan reported a dramatic increase in macrolide-resistant *M. pneumoniae*, and the macrolide resistance rate exceeded 90% in 2011 [16–19]. In South Korea, the prevalence of macrolide-resistant *M. pneumoniae* increased from 0 to 63% within 10 years [20, 21]. In China, the infection rate of macrolide-resistant *M. pneumoniae* has always been high, ranging from 69 to 100% [22–24] in recent years. Given the distinct increase in the prevalence of macrolide-resistant *M. pneumoniae*, it is crucial to detect macrolide-resistant *M. pneumoniae* in a timely fashion and facilitate targeted treatment adjustments for *M. pneumoniae*-induced infections.

The resistance mechanism employed by *M. pneumoniae* involves point mutations in domain V of the 23S rRNA gene. Specifically, mutations at positions 2063 or 2064 lead to high-level resistance, whereas mutations at positions 2067 or 2617 are associated with low-level resistance to macrolides [25, 26]. Furthermore, point mutations that occur in the L4 and L22 ribosomal protein genes seem to have no impact on resistance to macrolides in *M. pneumoniae* [22, 26]. Until now, many erythromycin ribosome methylation (*erm*) and efflux pump (*mef*) genes introduced via transposons or plasmids have been reported in many strains of pneumococci and enterobacteria [27], but these genes have still not been detected in *M. pneumoniae* [28]. The existence of any other macrolide resistance mechanisms of *M. pneumoniae* remains unclear and requires further study. Although early reports have found some potential correlation between macrolide-resistance and genotype in China, most type 2 isolates are macrolide-susceptible [22–24, 28, 29]. The data are relatively limited, and the association of the macrolide-resistance with genotype does not exist abroad reports [14, 26]. Therefore, macrolide-resistance and genotypes of *M. pneumoniae* need to do further research.

In this study, 81 *M. pneumoniae* isolates were obtained from 271 throat swabs that were collected between 2014 and 2016 in Beijing, China, to evaluate the prevalence of macrolide-resistant *M. pneumoniae* and characterize its mechanisms of resistance, and analyze the correlation between genotype and macrolide resistance observed in *M. pneumoniae* in China.

## Methods

### Study design

Microbiological testing was conducted at the Department of Communicable Disease Diagnostics of National Institute for Communicable Disease Control and Prevention, Chinese Center for Disease Control and Prevention. This study was approved by the Research Ethics Committee of the National Institute for Communicable Disease Control and Prevention, Chinese Center for Disease Control and Prevention, the Research Ethics Committee of the Beijing Centers for Disease Control and Prevention, and the Research Ethics Committee of the Beijing Children’s Hospital. The segment of the work based on molecular analysis was carried out at laboratory of National Institute for Communicable Disease Control and Prevention, Chinese Center for Disease Control and Prevention.

### *M. pneumoniae* isolates, culturing, and genomic DNA extraction

From January 2014 to December 2016, total 271 throat swabs were collected from the Beijing Centers for Disease Control and Prevention and Beijing Children’s Hospital. None of the patients were immunocompromised, patients with neutropenia, and patients receiving immunosuppressive chemotherapy were excluded. All specimens were collected from patients with respiratory tract infections according to clinical symptoms. Each throat swab was cultured in *Mycoplasma* selective liquid media (OXOID) at 37 °C. When the color of the media changed from red to yellow, approximately 0.1 ml of the suspension was transferred onto agar to subculture and purify the bacteria using the filtration-cloning technique for *M. pneumoniae* clinical isolates. Genomic DNA from each obtained isolate was extracted with the QIAamp DNA MINI kit (QIAGEN) using the protocol for blood and body fluids, and the identity of each isolate was verified using real-time PCR [30].

### Antimicrobial susceptibility testing of *M. pneumoniae*

Minimum inhibitory concentrations (MICs) of four antibiotics, including erythromycin, azithromycin, levofloxacin and tetracycline (Sigma), were determined via micro-dilution methods using SP4 broth (Remel). *M. pneumoniae* M129 (ATCC 29342) and ICDC P028 (Clinical isolate) were used as macrolide-susceptible and -resistant controls, respectively. The MIC operation was carried out according to the latest version of CSLI M43-A. Serial two-fold dilutions of each antibiotic prepared in SP4 broth containing about 10^5^ colony-forming units (CFU)/ml of *M. pneumoniae* were plated in 96-well micro-plates. The micro-plates were sealed with sterilized liquid paraffin oil and incubated at 37 °C for 5 days. Each antimicrobial susceptibility test was performed in triplicate. The MIC was determined as the lowest concentration of antimicrobial agent that induced a color change in the control media.

### Amplification and sequencing domains II and V of the 23S rRNA gene and the L4 and L22 ribosomal protein genes

Amplification of domains II and V of the 23S rRNA gene and the L4 and L22 ribosomal protein fragments were performed using the primers described previously [26]. Each reaction was performed in a final volume of 20 μl, containing 2 μl 10× Ex Taq Buffer (Mg^2+^ plus), 0.4 μM primers, 1.6 μl dNTP mixture (each 2.5 μM), 0.2 μl TaKaRa Ex Taq (5 U/μl), 1 μl of template DNA, and nuclease-free water to achieve a 20-μl final volume. The cycling conditions were as follows: 95 °C for 3 min, followed by 30 cycles of 95 °C for 30 s, 55 °C for 30 s, and 72 °C for 60 s with a final extension step of 72 °C for 5 min. All amplification products were sequenced bidirectionally by Sangon Biotech (Beijing) Co. Ltd.

### Duplex real-time PCR assay for genotyping of *M. pneumoniae*

Amplification of the genotype specific regions from all obtained isolates was performed with the duplex real-time PCR assay described previously [31]. Each PCR mixture was prepared in a total volume of 25 μl and contained the following per reaction: 12.5 μl Platinum Quantitative PCR SuperMix-UDG (Life Technologies-Invitrogen), 1.5 μl MgCl_2_(50 mM), 0.5 μM final concentration of each primer, 0.2 μM final concentration of each probe, 1.25 U Platinum Taq DNA polymerase(5 U/μl; Life Technologies-Invitrogen), 1 μl PCR nucleotide mix (10 mM), 5 μl nucleic acid extracted from each specimen, and nuclease-free water to achieve a 25 μl final volume. Real-time PCR for each target was performed in the CFX96 Real-time PCR Detection System (Bio-Rad, Hercules, CA, USA) under the following conditions: predenaturation at 95 °C for 2 min, followed by 45 cycles at 95 °C for 15 s and 56 °C for 15 s. The data were analyzed with the CFX Manager Software (version 2.1; Bio-Rad).

### MLVA genotyping and VNTR detection in *p1* gene

Extracted nucleic acids from all 81 isolates were used as the template for PCR amplification of the four loci selected for multilocus variable-number tandem-repeat (VNTR) analysis (MLVA), as described previously [32]. Furthermore, all nucleic acids were also as the template for PCR amplification of the high resolution VNTR sequence in the p1 gene [24], The PCR products were sequenced by the Sangon Biotech (Beijing) Co., Ltd.

## Results

### Clinical *M. pneumoniae* culture and identification

A total of 81 *M. pneumoniae* isolates were obtained from 271 patients. Of the 81 isolates, 27 were isolated in 2014, 19 were isolated in 2015, and 35 were isolated in 2016. Genomic DNAs from all 81 obtained isolates was identified by real-time PCR, which indicated that each isolate was indeed *M. pneumoniae*.

### Antimicrobial susceptibility of *M. pneumoniae*

Of the 81 clinical isolates, 53 (65.4%) were erythromycin resistant (MIC, ≥256 μg/ml). These erythromycin-resistant isolates, as well as the reference strain ICDCP028, also showed resistance for the azithromycin. The MIC for the 15-member macrolide, azithromycin (2–64 μg/ml), was lower than that of erythromycin. The other 28 isolates, as well as the reference strain, M129, were macrolide-susceptible with each having an MIC of ≤0.008 for erythromycin and azithromycin. All 81 clinical isolates were susceptible to tetracycline and levofloxacin used in this study. (Table 1).

**Table 1:** Genotype characteristics and MIC ranges of four antimicrobial agents used against 81 *M. pneumoniae* clinical isolates from 2014 to 2016

Year	Mutation in the 23S rRNA	Isolates number		MLVA genotype (Numbers)		No. of “AGT” VNTRs repeats in p1 gene (Number)		MIC (μg/ml)			
		type1	type2	type1	type2	type1	type2	ERY	AZM	LVX	TET
2014	A2063G	13	2	4/5/7/2 (10) 4/5/7/3 (2) 4/4/7/2 (1)	3/5/6/2 (2)	5 repeats(1) 7 repeats(6) 8 repeats(5) 11repeats(1)	6 repeats(1) 8 repeats(1)	≥256	2–32	0.25–1	0.016–0.25
	A2064G	1	0	4/5/7/2 (1)	0	7 repeats(1)		≥256	4	0.25	0.032
	None	2	9	4/5/7/2 (2)	3/5/6/2 (9)	5 repeats(1) 7 repeats(1)	6 repeats(3) 7 repeats(2) 8 repeats(3) 12 repeats(1)	≤0.008	≤0.008	0.25–1	0.032–0.25
2015	A2063G	12	2	4/5/7/2 (12)	3/5/6/2 (2)	4 repeats(1) 6 repeats(5) 7 repeats(4) 8 repeats(2)	8 repeats(2)	≥256	2–64	0.125–0.5	0.032–0.5
	None	0	5	0	3/5/6/2 (5)	0	5 repeats(1) 8 repeats(4)	≤0.008	≤0.008	0.25–1	0.032–0.25
2016	A2063G	19	4	4/5/7/2 (17) 4/5/7/3 (2)	3/5/6/2 (3) 3/5/7/2 (1)	6 repeats(2) 7 repeats(5) 8 repeats(4) 9 repeats(6) 10 repeats(1) 12 repeats(1)	7 repeats(3) 10 repeats(1)	≥256	2–64	0.125–1	0.016–0.5
	None	0	12	0	3/5/6/2 (11) 3/5/7/2 (1)	0	6 repeats(2) 7 repeats(5) 8 repeats(3) 10 repeats(1) 14 repeats(1)	≤0.008	≤0.008	0.25–1	0.032–0.5

The MIC of each agent was defined as the lowest concentration of each antibiotic that prevented the color change

*ERY* erythromycin, *AZM* azithromycin, *LVX* levofloxacin, *TET* tetracycline

### Clinical patient data

Clinical data for *M. pneumoniae*-related cases of pneumonia were available for the 81 patients, 53 (65.4%) of whom had pneumonia caused by macrolide-resistant isolates. None of patients had coinfection with *Streptococcus pneumoniae* (as defined by positive results of sputum culture, blood culture, and urinary antigen testing for *S. pneumoniae*) or *Legionella pneumophila* (as defined by urinary antigen testing). Demographic characteristics and clinical presentations were similar between the group infected with macrolide-resistant isolates (53/81) and the group infected with macrolide-susceptible isolates (28/81) (Table 2).

**Table 2:** Clinical data comparing 81 cases of respiratory tract infections caused by *M. pneumoniae* with different in vitro susceptibility to macrolides

	Macrolide-resistant *M. pneumoniae* group (*n* = 53)	Macrolide-susceptible *M. pneumoniae* group (*n* = 28)
Male	22 (41.5%)	11 (39.2%)
Clinical Symptoms		
Temperature (°C)	38.12	37.96
Cough	39 (73.6%)	18 (64.3%)
Sputum	33 (62.3%)	(53.8%)
Headache	16 (30.2%)	11 (39.3%)
Chest pain	3 (5.7%)	0
Leukocyte count (10^9^ cells/L)	8.44	8.71
Antibiotics usage before detection	14 (26.4%)	10 (35.7%)

### Amplification and sequencing of the 23S rRNA gene and the L4 and L22 ribosomal protein genes

98.1%(52/53) macrolide-resistant clinical isolates harbored the A2063G mutation in domain V of the 23S rRNA gene. Only one (1/53) macrolide-resistant clinical isolates harbored the A2064G mutation in domain V of the 23S rRNA gene. None 2063 or 2064 mutation was found in 28 macrolide-susceptible clinical isolates. Furthermore, no other mutations in domain V of the 23S rRNA gene were observed in the 81 isolates (Table 1). The C162A and A430G mutations in the L4 ribosomal protein gene and the T279C mutation in the L22 ribosomal protein gene were identified in 34 isolates, including the reference strain ICDC P028.

### Genotyping of *M. pneumoniae* with three methods

A total of 47 (58.0%) isolates were classified as type 1, and the other 34 (42.0%) isolates belonged to type 2 with duplex real-time PCR. Among the 47 type 1 isolates, 45 were macrolide-resistant, and only two was macrolide-susceptible. The 34 type 2 isolates, 8 were macrolide-resistant, and other 26 was macrolide-susceptible. 89.4% (42/47) type 1 isolates were 4/5/7/2 MLVA type, and 94.1% (32/34) type 2 isolates were 3/5/6/2 MLVA type (Table 1). The numbers of VNTR in the p1 gene from all 81 *M. pneumoniae* isolates were different, ranging from 5 to 14, as shown in Table 1.

### Macrolide-resistant isolates with genotyping from 2014 to 2016

The total Macrolide-resistant rate was 59.3, 73.7 and 65.7% from 2014 to 2016, respectively. However, the macrolide-resistant rate of type 1 isolates was 87.5%(14/16), 100.0% (12/12) and 100.0% (19/19), and was only 18.2% (2/11), 28.6% (2/7) and 25.0% (4/16) in type 2 isolate from 2014 to 2016 (Table 1).

## Disscussion

In general, macrolides are the primary therapeutic agent for treating *M. pneumoniae* infections in both children and adults, but the macrolide resistance rate has been shown to be very high in clinical isolates of *M. pneumoniae* in China in recent years [22–24, 31]. Basic clinical data demonstrated that the demographic characteristics, clinical presentations, and biochemical blood indices were similar between the group infected with macrolide-resistant isolates and the group infected with macrolide-susceptible isolates, which is consistent with our previous report [22]. This suggests that the pathogenicity of the macrolide-susceptible and macrolide-resistant isolates is unremarkable. However, our previous study found that patients infected with macrolide-resistant *M. pneumoniae* required significantly longer durations of antibiotic therapy and needed more time to recuperate from fevers [22]. Unfortunately, we did not obtain further clinical treatment data in this study. Although, Lluch-Senar [33] found that type 2 strains show higher expression levels of CARDS toxin, a protein recently shown to be one of the major factors of inflammation. It proposed that type 2 strains could be more toxigenic than type 1 strains of *M. pneumoniae*. Our basic clinical data still have not found some features in common for the patients with different genotypes. This suggests that the pathogenicity may also unremarkable for *M. pneumoniae* with genotypes. Therefore, the influence of the macrolide-resistance and the genotypes of *M. pneumoniae* isolates on the effect of the clinical outcomes requires further investigation.

Macrolide resistance in *M. pneumoniae* is strongly associated with mutations in the 23S rRNA gene [34]. Several common mutations in the 23S rRNA gene were found at positions 2063, 2064, and 2617 [15, 35, 36]. Among these genetic loci, the A2063G and A2064G mutations are responsible for high levels of macrolide resistance in *M. pneumoniae*. Notably, the A2063G mutation in domain V of the 23S rRNA gene is the most prevalent in macrolide-resistant *M. pneumoniae* isolates in China [22, 28, 31, 37, 38]. In this study, 98.1%(52/53) macrolide-resistant clinical isolates harbored this A2063G mutation. Only one (1/53) macrolide-resistant clinical isolates harbored the A2064G mutation, and no other mutations in the 23S rRNA gene were observed. These findings coincided with the results from the antimicrobial susceptibility tests. Based on our antimicrobial susceptibility results, each of the A2063G isolates was responsible for high-level resistance to erythromycin (≥256 μg/ml), and azithromycin (2–64 μg/ml) in vitro. The susceptibility data also revealed that all *M. pneumoniae* isolates were susceptible to tetracycline and fluoroquinolones. Taken together, these findings suggest that these antibiotics have the potential to be used as alternatives for treating *M. pneumoniae* infections in adults with cases of high macrolide resistance, but this class of antibiotics is not ordinarily recommended for children, except in particular cases.

Furthermore, mutations in the L4 and L22 ribosomal proteins are related to macrolide resistance in other species [39]. In this study, we found that the C162A and A430G mutations in the L4 ribosomal protein gene and the T279C mutation in the L22 ribosomal protein gene were observed in all isolates classified as type 2 for the p1 gene, which is consistent with our previous report [22]. An intensive study using whole genome sequence analysis of 20 Chinese clinically isolated strains (unpublished data) and *M. pneumoniae* genomes available in NCBI database [33, 40], identified the C162A, A430G, and T279C mutations in the ribosomal protein gene that were detected in all type 2 isolates. We reasoned that the three mutations in L4 and L22 were not responsible for macrolide resistance, but rather influenced the particular genotype of *M. pneumoniae*.

This study showed a comparatively low macrolide resistance rate of 65.4% in China, in recent 10 years [22–24, 37, 38, 41–45]. Interestingly, 95.7% (45/47) of the type 1 isolates were resistant to macrolides, whereas only 23.5% (8/34) of the type 2 isolates were resistant to macrolides in this study. To our knowledge, this interesting phenomenon has not been reported in other counties [26, 46]. Due to the relatively low resistance rate of type 2 isolates, the overall rate of macrolides resistance is lower than previously reported in China [23, 24, 28, 29, 37, 38, 47]. In recent publication, we found that *M. pneumoniae* type 1 was the predominate genotype present during 2008–2012 in Beijing, and a genotype shift from type 1 to type 2 began to occur in 2013. Here, the percentage of type 2 *M. pneumoniae* isolates was low to19.5% (34/349) before 2012, and was 26.3% (8/41) during 2012, but raised to 39.4% (29/66) during 2013–2014 [31]. In this study, genotyping results support this earlier research findings. The percentage of type 2 *M. pneumoniae* isolates was 42.0% (34/81) during 2014–2016. These indicates that the genotype of *M. pneumoniae* isolates in Beijing is shifting from type 1 to type 2. Based on earlier work [48], which reported that *M. pneumoniae* genotype shifts occur every 8–10 years and that a shift from one type to another requires 2–3 years. Our publication deduced that type 2 will likely become the predominate genotype in Beiijing over the next few years [31]. However, in this research, the *M. pneumoniae* genotype in Beijing still did not become the absolute dominant type 2 till 2016, but entered the stage where the ratio of type 1 and type 2 was nearly closed. This is not consistent in the *M. pneumoniae* genotype shift cycle reported in publication [48]. Maybe the genotype shift cycle takes more longer in Beijing, and perhaps there is no very regular periodic genotype shifting about *M. pneumoniae* [14, 26, 49]. After all, *M. pneumoniae* genotyping research is less than 15 years in China, and requiring researchers to do longer-term monitoring and research.

Although the macrolide resistance rate of type 2 *M. pneumoniae* isolates is not very high in this study. Compared with the previous data, we found that the macrolide resistance rate of type 2 isolates is also increased synchronously with the increase of the proportion of type 2 isolate (Table 3). No macrolide resistance *M. pneumoniae* of type 2 isolates was found in 2008 in Beijing. The proportion of macrolide resistance *M. pneumoniae* of type 2 isolates increased from 15.6% before 2012 to 23.5% in this study. Macrolide resistance rate of type 2 *M. pneumoniae* increased by 50.6% within 9 years in China. In contrast, although the proportion of type 1 isolates is decreasing gradually, the macrolide resistance rate still remains above 90.0%. We deduced that this phenomenon may be related to the irregular use of antibiotics in China. The widespread overuse of antibiotics is very common in China, especially in some district hospitals. Under tremendous antibiotic selection pressure, almost all of the predominant type 1 *M. pneumoniae* strains isolated in China could certainly turn into macrolide-resistant isolates. Relatively speaking, the inferior type 2 *M. pneumoniae* strains with little chance to suffer from antibiotic selection pressure might still remain macrolide-susceptible in early years. With the increasing of the proportion of type 2 isolates in recent year, the chances of exposure to macrolide antibiotics increase greatly. Under the excessive selection of antibiotic pressure, macrolide -resistant type 2 isolates appear and spread rapidly, resulting in an increase in the macrolide -resistant rate of type 2 isolates in recent years in Beijing. The MLVA and the VNTRs in the p1 gene results both indicated that the increase of resistance in genotype 2 is not due to the spread of same clone. We will continue to monitor the status of the genotypes and macrolide resistance rates of *M. pneumoniae* in Beijing over the next few years to verify our hypotheses.

**Table 3:** Prevalence of macrolide resistance and genotyping in *M.pneumoniae* clinical isolates in China

Area	Year	Macrolide resistance rate (No. of resistant isolate/total isolate)	Type 1 macrolide resistance rate (No. of type 1 resistant isolate/total type 1 isolate)	Type 2 macrolide resistance rate (No. of type 2 resistant isolate/total type2 isolate)	References
China ^a^ (Beijing, Shanghai)	2008–2011	86.0% (351/408)	91.8% (345/376)	15.6% (5/32)	See below
Beijing, China	2008–2009	68.7% (46/67)	78.0% (46/59)	0% (0/8)	[22]
Shanghai, China	2008–2009	90.0% (90/100)	95.7% (89/93)	14.3 (1/7)	[46]
Beijing, China	2011	95.0% (38/40)	95.0% (38/40)	NA	[43]
Beijing, China	2008–2011	88.1% (177/201)	93.5% (172/184)	23.5% (4/17)	[24]
China (Beijing)	2014–2016	65.4% (53/81)	95.7% (45/47)	23.5% (8/34)	This study

^a^References with consistent Macrolide resistance and genotype included

*NA* No type2 isolates

Our study had limitations. This was a single-center study conducted over 3 year, and only 81 *M. pneumoniae* isolates were studied. The statistical power may have been insufficient to assess other clinical outcomes.

## Conclusions

In summary, macrolide-resistant *M. pneumoniae* in Beijing, China, has been present at high levels in recent years. The A2063G transition in domain V of the 23S rRNA gene was identified in all macrolide-resistant *M. pneumoniae* isolates, and we noted that the mutations in L4 and L22 were not responsible for macrolide resistance but rather influenced the particular genotype of *M. pneumoniae*. We deduced that the significant increased macrolide-resistant type 2 isolates may be due to the genotype shifting of *M. pneumoniae* in Beijing. The relationship between genotype shifts and macrolide resistance in *M. pneumoniae* needs to be further verified with more extensive surveillance data.

## Abbreviations

AZM: Azithromycin
CARDS TX: Community-Acquired Respiratory Distress Syndrome Toxin
ERY: Erythromycin
LVX: Levofloxacin
MIC: Minimum inhibitory concentration
MLVA: Multilocus variable-number tandem-repeat analysis
TET: Tetracycline
VNTR: Variable-number tandem-repeat
